# Group-based PFMT programme for preventing and/or treating UI in pregnant women: protocol of a randomized controlled feasibility study

**DOI:** 10.1186/s40814-023-01410-2

**Published:** 2023-10-31

**Authors:** Xiaowei Yang, Aixia Zhang, Rong Zhu, Lynn Sayer, Sam Bassett, Sue Woodward

**Affiliations:** 1 Department of Florence Nightingale Faculty of Nursing, Midwifery and Palliative Care, King’s College London, London, UK; 2 Department of Clinical Teaching and Research, Nanjing Vocational Health College, Nanjing, China; 3 Nursing Department, Nanjing Maternity and Child Health Care Hospital, Nanjing, China

**Keywords:** Group-based intervention, Pelvic floor muscle training, Urinary incontinence, Pregnant women

## Abstract

**Background:**

Urinary incontinence (UI) is a prevalent health problem in women worldwide. Many women experience UI during pregnancy. The National Institute for Health and Care Excellence (NICE) recommended pelvic floor muscle training (PFMT) as the first-line conservative treatment for UI. However, it is not widely implemented due to the limited number of healthcare trainers. Group-based PFMT has been used with older women and a limited number of maternity studies. But the effectiveness of the group-based PFMT needs to be investigated because the overall quality of the studies is low. Therefore, this study aims to assess the feasibility of delivering a group-based PFMT programme for pregnant women in Nanjing city.

**Methods:**

This feasibility study will be conducted in Nanjing Maternity and Child Health Care Hospital in China, using a mixed methods design to investigate the feasibility and acceptability of delivering group-based PFMT to pregnant women. Pregnant women with or without the symptoms of UI will be included. This study aims to recruit 48 pregnant women with 24 in each arm. Participants will receive either the group-based PFMT delivered by a midwife or usual antenatal care which includes only verbal instruction on PFMT. The study will assess the completion rates, acceptability of outcome measures, recruitment and retention rate and calculate an appropriate sample size for a future study.

**Discussion:**

The results of this study will inform the design and implementation of a definitive randomized clinical trial to explore the effectiveness of the intervention.

**Trial registration:**

ClinicalTrials.gov, NCT05242809.

**Supplementary Information:**

The online version contains supplementary material available at 10.1186/s40814-023-01410-2.

## Background

Urinary incontinence (UI), one of the most prevalent health problems in women worldwide, is defined as a complaint of involuntary loss of urine [1]. Many women experience UI during pregnancy, and the prevalence of UI peaks in the third trimester [2]. Physiological changes occur during pregnancy including increased abdominal pressure and progesterone levels. Along with injury to pelvic floor muscles during vaginal delivery, the strength and endurance of pelvic floor muscles may be damaged which make women more susceptible to UI [3, 4].

The National Institute for Health and Care Excellence (NICE) recommended conservative treatment for the management of UI, for example, lifestyle interventions, physical therapies including pelvic floor muscle training and electrical stimulation, behavioural therapies and neurostimulation [5]. In addition, researchers explored other approaches to provide more conservative therapeutic options for UI, for example, vaginal cones [6], pelvic floor electromyography biofeedback [7] and magnetic stimulation [8] although these options have not been widely used and recommended.

Individualized pelvic floor muscle training (PFMT) is recommended as a first-line conservative treatment for stress urinary incontinence (SUI) and any type of UI in the clinical practice guidelines [9]. However, PFMT is not implemented in many countries often due to a limited number of suitably skilled health professionals [10–12]. In order to provide PFMT to more women and overcome the human resource barriers, there is a need to explore methods of implementing PFMT at scale.

Recent systematic reviews showed that group-based PFMT was effective in preventing and treating UI in pregnant women [13, 14]. Group-based approaches have been employed within health promotion for many years and found to be an economical way to facilitate behaviour modification [15]. In addition, group-based interventions are reported to foster peer support, reduce stigma and isolation feelings and encourage active self-management [16]. It was reported that group-based training has a positive impact on self-management which could facilitate adherence to PFMT and support women to undertake the exercise in the long term [17]. However, a limited number of studies were found to assess the effectiveness of group-based PFMT in pregnant women [13]. In addition, the overall quality of the included studies was assessed to be low based on the Grading of Recommendations, Assessment, Development and Evaluations (GRADE) [13].

Well-designed randomized controlled trials are needed to provide evidence of the effectiveness of group-based PFMT in pregnant women. According to the Medical Research Council (MRC) framework, feasibility or pilot studies should be conducted before a full-scale randomized controlled trial to investigate the acceptability of an intervention, estimate the likely rates of recruitment and retention and calculate appropriate sample sizes [18]. In addition, sample characteristics, adherence and identification of effective recruitment approaches can be assessed through a feasibility study. Feasibility studies can help to avoid human and financial research waste in a large RCT [19]. Therefore, this paper describes the protocol for the feasibility testing of a group-based PFMT programme in pregnant women in Nanjing city in China before evaluating the effectiveness within a fully powered RCT.

## Research question

Is it feasible to deliver a group-based PFMT programme to pregnant women in Nanjing city in China?

## Method

### Aims and objectives

This study aims to test the feasibility and acceptability of the intervention in preparation for a full-scale randomized controlled trial. The objectives of the study are as follows:

1. To explore the feasibility and acceptability of the group PFMT intervention in pregnant women in Nanjing city in China
2. To estimate the recruitment and retention rates for a group-based PFMT supervision programme
3. To assess the attendance and adherence within the supervised PFMT programme
4. To assess the acceptability of proposed outcome measures
5. To conduct a process evaluation through post-intervention interviews with the participants and the midwife implementer to consider intervention fidelity and refine the programme for future trial

### Study design

**Fig. 1:**
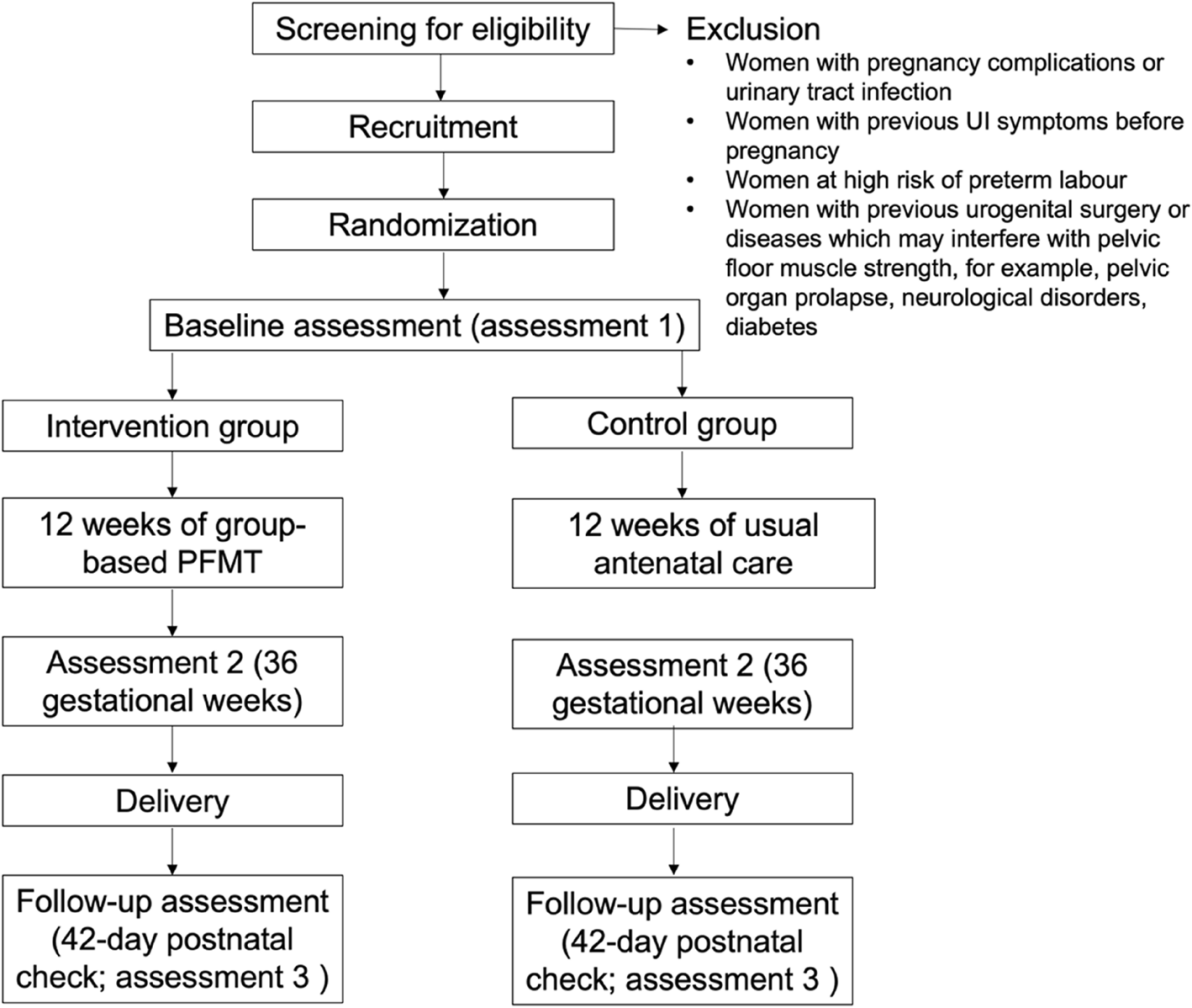
Flow chart of the feasibility study

This single-centred, three-phased study uses a mixed methods research design guided by the MRC [18] and behaviour change wheel (BCW) frameworks [20]. The use of these validated frameworks within the feasibility study will be published separately in a co-design paper. This trial will comprise a two-arm feasibility RCT, in which participants will be randomly allocated to either group-based PFMT or usual antenatal care. Participants will be assessed at three time points during the study: baseline (24 gestational weeks), post-intervention (approximately 36 gestational weeks) and follow-up assessment (42 days after delivery). This study has been approved by the ethics committees of King’s College London (LRS/DP-21/22–26,714) and Nanjing Maternity and Child Health Care Hospital (2021-KY-084) and has been registered with ClinicalTrials.gov under NCT05242809. This study has been designed following the Standard Protocol Items: Recommendations for Interventional Trials (SPIRIT) [21] and reported according to the Consolidated Standards of Reporting Trials (CONSORT) extension for pilot and feasibility trials statement [22]. The SPIRIT checklist is presented as the Additional file 4: Table S1. The study flow diagram is presented in Fig. 1.

The study comprises three phases: co-design of the group-based PFMT programme (phase 1), feasibility testing of the group-based PFMT programme (phase 2) and a process evaluation (phase 3). The process of the co-design of the group-based PFMT programme is in preparation for publication elsewhere, and the protocol for phases 2 and 3 is reported in this paper.

### Setting and sampling

The feasibility of two-arm RCT will be conducted in the antenatal clinic at Nanjing Maternity and Child Health Care Hospital in China. It is one of the biggest maternity hospitals in Nanjing city, where 50 to 80 women at around 19–20 gestational weeks attend antenatal care appointments each day following their referral to the hospital for their maternity care. Nanjing city is the capital city of Jiangsu province and is a well-developed city in China [23]. The prevalence of UI in pregnant women in this city was reported to be 37.8% in 2020 [24]. The selection of enrollment at 24 gestational weeks is based on the discussion in workshops with stakeholders and evidence. Pregnant women in their 19–20 gestational weeks need to be referred to a general hospital or an obstetric hospital from community hospitals in China. It is not possible to provide the group-based PFMT programme to the same group of women for consecutive 3 months until they are referred to a general hospital or an obstetric hospital. In consideration of the feasibility of this study, the stakeholders discussed not increasing the number of pregnant women’s visits to the hospital. Therefore, this study started recruiting pregnant women on their first visit to the obstetric hospital and obtaining their consent on participating in the group-based PFMT programme, then started providing face-to-face supervision on their next antenatal check (around 24 gestational weeks) and support them to perform this exercise at least 3 months in their pregnancy which is recommended by the NICE.

### Sample size

As this is a feasibility study, formal sample size calculation has not been performed. The sample size for a feasibility study needs to meet a balance between not being too large, which may cause a burden on health professionals and participants, and also not being too small as the consent rate, attrition and sample size for a large-scale RCT need to be calculated. Based on the recommendation from the National Institute for Health Research [25], an overall sample size of 30 or greater was considered sufficient for a feasibility study. In addition, a sample size between 24 and 50 is sufficient to calculate a standard deviation of an outcome, which can then be used for a formal calculation for a full-scale RCT [26, 27]. A study by Whitehead et al. [28] developed a method for determining the best approach to calculate the required sample size for a pilot trial and found that for studies where standardized differences are large, to achieve 90% power in the main trial, the required sample size for each arm is 10. For women receiving nonsurgical therapies for incontinence, reductions of four to six points in the International Consultation on Incontinence Questionnaire-Urinary Incontinence Short Form (ICIQ-UI SF) are needed to show clinically meaningful [29]. Two further studies consider the attrition rates in group-based pelvic floor muscle training in pregnant women or postpartum women range from 8.57 to 53.33% [30, 31]. Therefore, this study aims to include a sample size of 48 participants with 24 in each arm.

### Eligibility criteria

This study will target pregnant women meeting the inclusion criteria attending the antenatal clinic in Nanjing Maternity and Child Health Care Hospital without considering whether they have symptoms of UI during pregnancy.

The following are the inclusion criteria:

1. Nulliparous women who are aged 18 years and older
2. Gestational ages of 19–24 weeks
3. With or without the symptoms of UI
4. Singleton foetus
5. Capable of giving valid informed consent

The following are the exclusion criteria:

1. Women with pregnancy complications or urinary tract infection
2. Women with pre-existing UI symptoms before pregnancy
3. Women at high risk of preterm labour
4. Women with a history of previous urogenital surgery or diseases that may interfere with pelvic floor muscle strength, for example, pelvic organ prolapse, neurological disorders and diabetes

### Recruitment, consent, assessments, randomization and allocation concealment

The principal researcher will provide details of the study to the midwives and nurses who run the Nanjing Maternity and Child Health Care Hospital antenatal clinics. The midwives and nurses will be responsible for giving information about this study to the women. The midwives and nurses will screen potential participants based on the inclusion criteria to determine whether they are eligible to take part in the study when they attend the antenatal clinics at around 19–20 gestational weeks. If the participants are eligible to take part in the study, the midwives and nurses will provide participants with an information sheet. The midwives and nurses will be trained before speaking to women to assess eligibility for the study, and the midwives and nurses will inform the potential participants that the questions are asked only for assessing whether they are eligible for the study; this information will not be recorded in their medical records. Potential participants will be informed that choosing not to take part in or withdrawing from the study will not affect the care, treatment or service they receive in any way. They may also choose not to take part in the study or withdraw from the study at any time without giving a reason.

Potential participants will not need to tell the midwives and nurses whether they want to take part in the study at the time. They will take the information sheet home to consider whether to take part in the study or not before they attend their next antenatal clinic appointment at around 23–24 gestational weeks. If the participants are willing to participate in the study, they will be asked to contact the principal researcher using the contact information on the information sheet before their next antenatal appointment.

Any woman who expresses interest will be telephoned by the principal researcher. The principal researcher will ensure all the participants understand the detailed study information they have been given. They can also discuss the study and any questions they might have with the principal researcher. Following the call, they will still have time to consider whether to participate in the study before their 24 gestational week antenatal appointment. If they agree to participate in the programme, they will be scheduled a date before 24 gestational weeks to meet the principal researcher, and written informed consent will be obtained. If the participants withdraw their interest before providing consent, recruitment will continue until there are enough potential participants. The time for recruitment and randomization will be extended to ensure enough participants are recruited for the study. After the participants have signed the consent form, they will meet the research midwife in another office in the hospital and complete the baseline data.

The participants will be randomly allocated to the usual antenatal care or group-based PFMT programme by using a computerized block randomization programme which is generated by the principal researcher (the size of the block is 16). Allocation will be concealed by using sealed opaque envelopes. The envelope will be offered to the participants in sequence by the midwife. They will complete the baseline assessment after randomization. The participants who are randomized to the group-based supervision group will then be scheduled a date by the midwife for the first group training session. The flowchart of the feasibility study and the flow for participants is presented in Additional file 2. According to the inclusion and exclusion criteria, 75% of the pregnant women are estimated to be eligible [32, 33]. Therefore, the recruitment is expected to be completed within 3 months, and the screening of the pregnant women will stop when 48 participants have been recruited. No reimbursement was provided for women consenting to participate or continue participating in this study.

### Blinding

It is not possible for the midwife delivering the intervention to be blinded to the pregnant women in the intervention groups or the usual antenatal care group. Also, the principal researcher will not be blinded to the participants to be invited in the interviews post-intervention. However, the principal researcher, who will be conducting baseline and follow-up data analysis, will be blinded to the allocation when analysing the feasibility data.

### Interventions

The participants in the intervention group will receive PFMT supervision in groups of eight women. The intervention will include verbal instruction on PFMT, the use of models and pictures of the pelvic floor to help women identify their pelvic floor muscles and then guide them on how to contract the pelvic floor muscles correctly. The content of this PFMT programme was adapted from a published protocol [34] and co-designed with stakeholders to suit the needs of a different population (pregnant women) and setting (Nanjing city) as recommended by the MRC framework [18]. The stages in co-designing the group-based PFMT programme are described in Additional file 2. In brief, the exercise includes three repetitions with 2-min rests between each repetition. One repetition comprised eight contractions with each held for 6 s, followed by three or four fast contractions. Learning and practising the exercise will be supervised in the group sessions and will be required to be repeated twice per day at home between group sessions.

All the group-based training sessions in the programme will be provided by one midwife to ensure consistency. The midwife will be trained beforehand by the principal researcher about the PFMT programme. The participants will be required to meet the midwife for group supervision once a month on the day of their regular antenatal check for consecutive four consecutive months (approximately 24 gestational weeks, 28 gestational weeks, 32 gestational weeks and 36 gestational weeks). The group supervision sessions will last for about 45 min which include attendance registration and progress review (5 min), education on the causes of UI and PFMT (10 min), skills training in sitting and standing positions (20 min), discussion on what is taught (8 min) and reminder about the next session (2 min). The registration of attendance and the training diary which will be provided to the participants after each session will be monitored to record adherence to the programme. Women who attend at least 75% of the supervision sessions will be considered as adherent. Motivation to maintain PFMT will be emphasized by the midwife and supported by WeChat groups which will be limited to the eight pregnant women in the group session. A reminder to practise PFMT as well as the audio instructions on doing PFMT will be sent automatically in the WeChat group twice a day. In addition, leaflets on PFMT, training diaries and stickers which motivate the participants to undertake PFMT will be provided. The PFMT leaflets and training diary were provided as Additional file 3. One day before the next session, a reminder of the session date will be sent to the WeChat group. The participants will be encouraged to discuss any topics related to PFMT and UI in the WeChat group to facilitate peer support. The midwife and the principal researcher will adopt administrative roles and monitor the WeChat groups but will not answer questions or participate in discussions. Any problems which the pregnant women encountered during the exercises will be addressed during face-to-face supervision. There will be ground rules set for the groups, for example, the women in the groups need to keep the information confidential within the group and treat each woman in the group with respect. If any women break the ground rules or discuss information in the WeChat group that is unsafe, the principal researcher will remind the women about the ground rules and correct any information that is dangerous. During the development of this study, the barriers identified from the systematic review [13], survey [24] and discussion with stakeholders, such as time commitment, forget to continue PFMT were mapped to the COM-B model and explored in a separate study. For reasons of equipoise, women in the comparator group will be offered the group-based PFMT intervention at the end of the study if they wish.

### Comparator

The comparator is usual antenatal care, which means the participants will receive verbal instruction on PFMT from midwives without any further supervision. The participants receive instructions on standard antenatal practice, such as eating, sleeping, nursing newborn babies for the purpose of preparing for delivery and the postnatal period. The participants will not be discouraged from performing PFMT for ethical reasons.

### Measures

The outcomes will involve both qualitative and quantitative outcome measures. One focus group interview with the participants and one semi-structured interview with the midwife who delivered the intervention will be held by the principal researcher in an office in the antenatal clinic. The midwife will not be involved in the focus group interview with the participants. The focus group interview aims to assess the acceptability of randomization, the outcome measure and the intervention. About six to eight participants will be purposively sampled from the intervention groups which means the participants were selected based on the researcher’s own judgement. The principal researcher aimed to select participants including both women who strongly adhered to the training programme and women who did not attend at least three face-to-face sessions. Compared to the other sampling approach, purposive sampling can better match the sample to the aims and objectives of the research [35]; however, it may bring a source of bias into the research which needs to be discussed in the interpretation of the interview when the feasibility study is written up [36]. The participants will be asked to discuss their experience and acceptability of the randomization process, the group-based PFMT, the UI outcome measure which is proposed for use in the future trial and their suggestions for future PFMT interventions. Discussion between participants will be encouraged. The consent for the interview will be obtained along with the consent for participating in the programme.

The semi-structured interview with the midwife aims to assess the acceptability of delivering the intervention. The midwife will be asked to discuss her experience of delivering the intervention, challenges and views of the feasibility of implementing the group-based PFMT across a larger population in Nanjing city. The interview will be conducted by the principal researcher in one office in the antenatal clinic. Both interviews will be digitally audio-recorded and transcribed verbatim.

The quantitative outcome measure will be recruitment rates and retention of participants, as well as the completion rates of UI outcome measures. The detail of outcome measures is shown in Table 1.

**Table 1:** Details of evaluation measures

Outcome measures	Evaluation measures
Recruitment and eligibility	Number of participants recruited
	Percentage of eligible participants
	Ambiguities regarding eligibility criteria
	Reasons for ineligibility
Data collection	Numbers of missing items in the collected questionnaires
Attrition	Rates of dropout
Resources needed to complete the study and the intervention	Length of time required for participants to complete questionnaires, interviews and exercise diary
Participant adherence	Numbers of attended sessions
	Numbers of exercise at home
Participants’ acceptability and satisfaction with intervention	Reasons for poor attendance
	Experience of attending the intervention

Besides the outcome measures which assess the acceptability of the programme, the UI outcome measures include self-reported UI and UI severity will be assessed by ICIQ-SF (a validated questionnaire which both assesses the severity of urinary loss and quality of life impact) [37]. The adherence to the programme will be assessed by attendance records from the group-based training sessions and completion of a training diary which includes the frequency and duration the participants self-report doing the exercises. The number of PFMT exercises at home was recorded in the training diary to assess participants’ adherence to home training. The training diary will be submitted to the principal researcher after the training sessions.

**Fig. 2:**
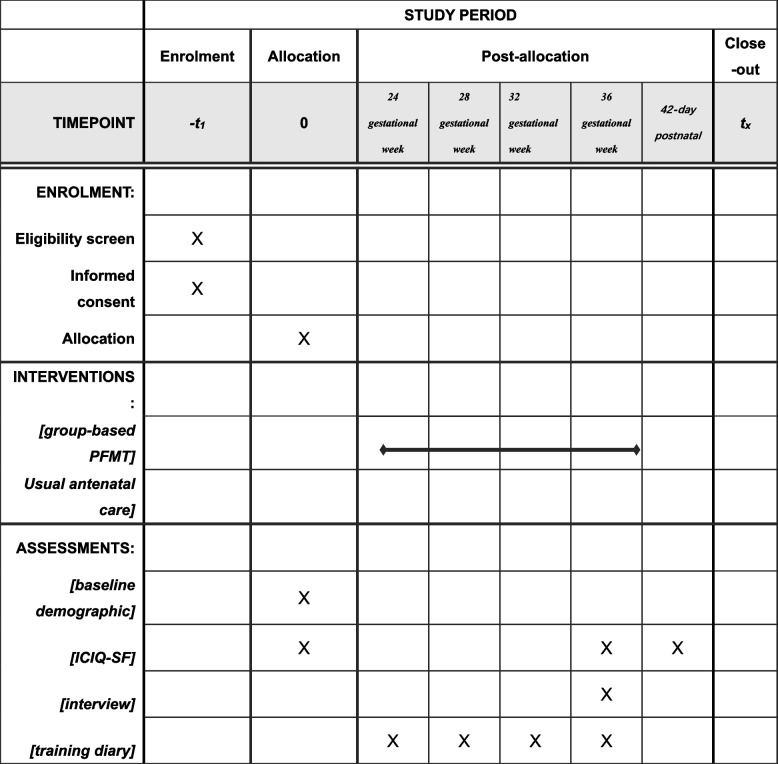
The schedule of enrolment, interventions and assessments

The UI outcome measure has been reviewed and approved by stakeholders (phase 1) and will take a total estimated completion time of 5–10 min on each occasion. Baseline outcome measures include demographics (name, date of birth, education level, body mass index and contact details) and will be collected after obtaining informed consent from the participants. The schedule for assessments is shown in SPIRIT figure [21] (Fig. 2).

For the feasibility and acceptability outcomes, the assessment and progression criteria are summarized in Additional file 4: Table S1. The progression criteria provided below are based on the evidence [38, 39], results from the systematic review by Yang et al. [24] and discussion with the stakeholders. The recruitment progression criteria to proceed with RCT was set at ≥ 50% of eligible participants consented to participate in the study in 3 months which was decided based on the findings from the survey. The retention progression criteria to proceed with RCT were set at ≥ 83% (40/48) of the participants submitting the baseline questionnaires, while ≥ 79% (38/48) of the participants submit at the first follow-up time point and ≥ 75% (36/48) of the participants submit at the second follow-up (42-day after delivery). The adherence progression criteria need to be assessed from two perspectives including ≥ 75% (18/24) of the participants complete at least 75% (3/4) of the face-to-face sessions and ≥ 75% (18/24) of the participants perform PFMT twice a day at home and record it on the training diary. The progression criteria for the acceptability and delivery of the intervention were the participants and the midwife had strongly positive views on their participation in the intervention. The feasibility study will proceed with a RCT if the above progression criteria are met. The progression criteria for proceeding with changes and stopping to proceed to a further study were provided in detail in Additional file 4: Table S1.

### Withdrawal of the study participants

Participants may withdraw from the study at any time without giving a reason. However, the data which has been collected will be kept up to the point of withdrawal. A participant will be withdrawn if they are lost to follow-up or request to leave the study or have premature delivery before 36 weeks gestation. If the participants in the supervised training group do not attend the face-to-face sessions without a reason, they will be considered as withdrawn.

### Adverse events report

Although there is no report on the adverse events of PFMT, any adverse events or unintended effects of trial interventions will be collected and reported to the principal researcher and the Research Ethics Committee.

### Data analysis

#### Process evaluation

**Fig. 3:**
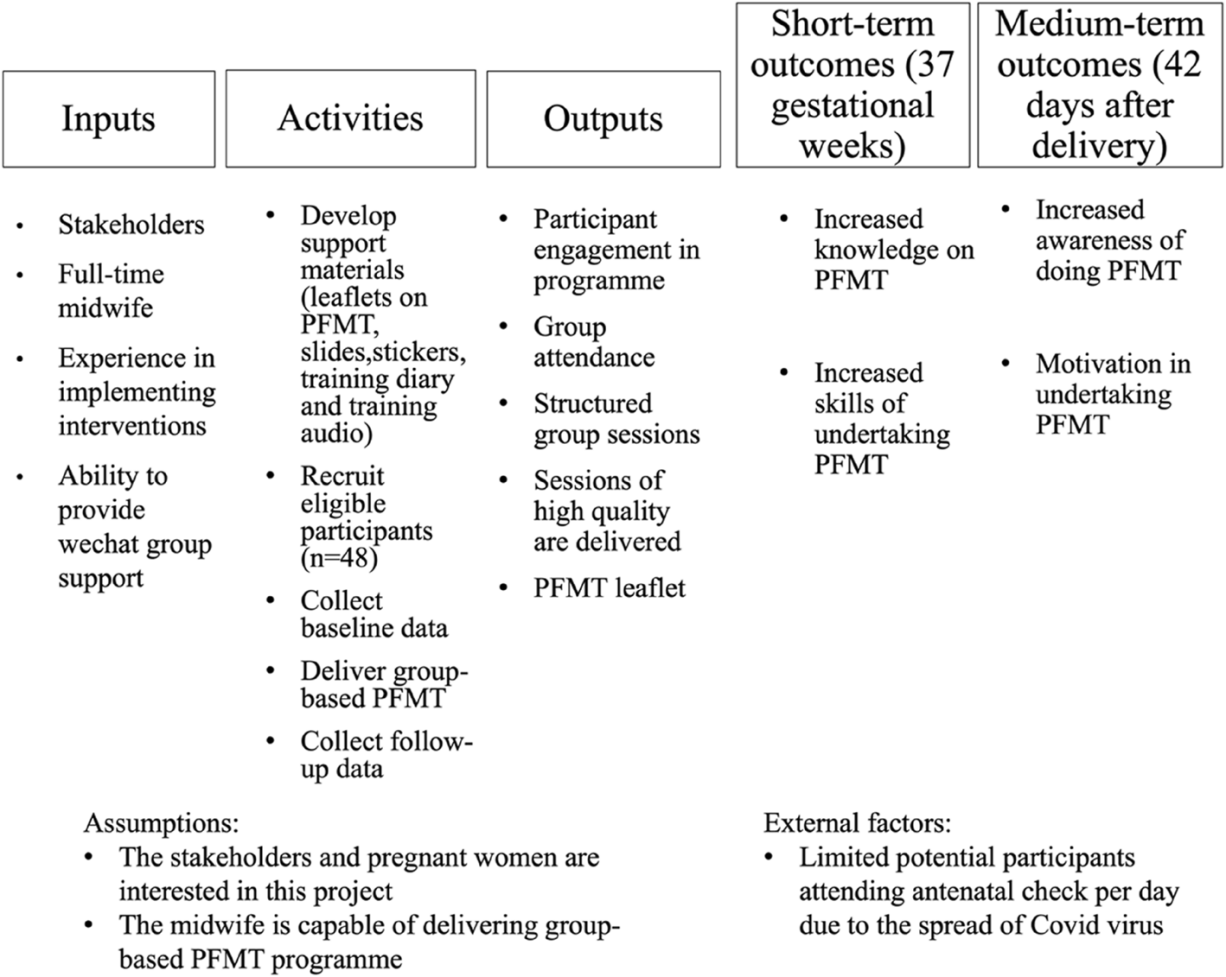
Logic model of the group-based PFMT programme

The process evaluation will be conducted based on the logic model (Fig. 3) for the intervention that was developed with stakeholders. The logic model will be used to monitor intervention fidelity and provide insight into how the intervention did or did not work in practice, identify any unintended consequences and refine the design of the future trial. It is useful for evaluation because it can help to optimize and structure data collection and analysis to explore the main aspects of an intervention and relationships between them [40]. The logic model can be used, for example, to evaluate the questions based on the programme, the information to answer the questions, the time of the collecting data and the data collection methods [41].

#### Analysis of quantitative data and qualitative data

Outcomes of the number and proportion of pregnant women who are screened and then eligible for the study, the number and proportion of pregnant women who consent to participate in the programme and be randomized to different groups and the number and proportion of pregnant women who completed outcome measures will be analysed using descriptive statistics. Group comparisons in UI outcome measures will be analysed using the Mann–Whitney test and chi-square test and presented with confidence intervals to evaluate evidence of the promise of the intervention. As this is a feasibility study, the analysis between groups is conducted only to obtain standard deviations of quantitative outcome measures of UI and to estimate sample size for the definitive RCT rather than aiming to show a definitive difference between the group-based PFMT group and the control group.

Qualitative data from the interviews will be analysed using pragmatic thematic analysis [42] to explore an in-depth understanding of the proposed programme, i.e. what are the experiences of participating in the intervention that may affect the feasibility of the intervention and future trial design. Thematic analysis allows potential themes from the transcripts to be identified without trying to fit the transcripts into one pre-existing coding frame. Therefore, this method is applied in many qualitative studies and is considered to be accessible and data-driven [42].

#### Data management and confidentiality

The approach to data management follows the requirements of King’s College London. Audio data which is collected during the interviews will be recorded using an encrypted digital recording device and stored securely on a password-protected King’s One Drive site. Recordings will then be deleted from the recording device. After the recordings have been transcribed, the recordings will be deleted from King’s One Drive site. The pregnant participants’ names will be converted into numbers which cannot be linked to the person to ensure anonymity. The date of birth and biographical data will be stored under the numbers. Hard copy data including paper questionnaires and training diaries will be stored in a locked cabinet in the principal investigator’s locked office.

Only the midwife’s experience in delivering the group-based PFMT programme will be identifiable and pseudonymized because she will be the only one who delivers the programme, so her experience cannot be anonymized. However, the data from the midwife will not be published and no identifiable data will be included in research publications. The data from the midwife’s interview will help to refine the protocol for a future full trial.

## Discussion

This study aims to assess the feasibility of implementing a theory-based PFMT programme in a group format with pregnant women. This study will support the design of a definitive RCT with adequate power examining the effectiveness of group-based PFMT in preventing and treating UI in pregnant women. The methods of this study will be modified in a future trial if there are any qualitative or quantitative indicators of problems with acceptability and feasibility that have an impact on recruitment, retention, randomization or fidelity. If there is a significant modification, a pilot study with clear stop/proceed rules will be considered prior to a full trial. The data will not be used to assess the effectiveness of the group-based PFMT programme within this feasibility study.

The strengths of this study include the group-based PFMT programme having been co-designed with stakeholders based on behaviour change theory which will facilitate adherence to the training programme. In addition, this study will investigate the facilitators and potential barriers to participating in and delivering a group-based PFMT programme from both participants’ view and the healthcare provider’s view. A limitation is that there will only be one midwife responsible for delivering group-based PFMT. This may not simulate real clinical practice and lack of experience in delivering group-based PFMT programmes by different health providers. Another limitation is this study only included nulliparous women which may limit the generalizability of the findings in all pregnant women. If a group-based PFMT programme in pregnant women in Nanjing city is shown to be feasible and effective, this will offer support for the future definitive trial.

## Trial status

The trial had not started recruiting when submitted to the journal.

### Supplementary Information

**Additional file 1.** SPIRIT Checklist.

**Additional file 2.** Feasibility study flow chart.

**Additional file 3.** PFMT leaflets and training diary.

**Additional file 4:** **Table S1.** Feasibility outcomes and progression criteria.

## Data Availability

The datasets used and/or analysed during the current study will be available from the corresponding author upon reasonable request.

## Abbreviations

BCW: Behaviour change wheel
BCT: Behaviour change techniques
CONSORT: Consolidated Standards of Reporting Trials
MRC: Medical Research Council
NICE: National Institute for Health and Care Excellence
PFMT: Pelvic floor muscle training
RCT: Randomized controlled trial
SPIRIT: Standard Protocol Items: Recommendations for Interventional Trials
SUI: Stress urinary incontinence
UI: Urinary incontinence
