# Supplementary material for: Group-based PFMT programme for preventing and/or treating UI in pregnant women: protocol of a randomized controlled feasibility study
Source: Pilot Feasibility Stud. 2023 Oct 31;9:180. doi: 10.1186/s40814-023-01410-2 (PMC10617193; doi:10.1186/s40814-023-01410-2)
Supplement: Supplementary file 2 — Additional file 2. Feasibility study flow chart. [file 40814_2023_1410_MOESM2_ESM.docx]

Supplementary Figure 1: Flowchart of study Phase 1

A logic model was developed during the stakeholder group meetings, and it will be used to monitor intervention fidelity and provide insight into how the group-based intervention work in practice.

Publish (1) how the intervention programme was designed following the stakeholder group meeting and behaviour change wheel.

(2) the protocol of the feasibility study

Simulation of the whole programme

**Stakeholder workshop 4**: reviewed the training programme and select BCTs according to APEASE criteria

**Stakeholder workshop 3:** reviewed pelvic floor muscle training regimen with the stakeholders and discussed the facilitators which may encourage the pregnant women to adhere to the programme and how to overcome the barriers which stop pregnant women participating in the programme.

**Stakeholder workshop 2** the outcomes from the first meeting and the protocol which was developed by Bo et al (1999) and was employed in previous studies of group-based PFMT was sent to all the members in the stakeholder development group

**Stakeholder workshop 1** the principal researcher introduced the members in the stakeholder group and presented the documents which have been sent to the members with a slide when meeting starts.

Recruit stakeholder group members:

four pregnant women with urinary incontinence (UI), four pregnant women without UI, two midwives, two physiotherapists.

**PHASE 1:**

**Development of the group-based PFMT programme**

**Activities with stakeholders**

The principal researcher draft implementation and training plans, develop training and supporting materials.

Selecting support approach for women to adhere to the programme need further discussion.

The duration of the programme, frequency, session number, session duration, numbers of women in a group, any support to the pregnant women of group-based supervision, and the detail of home exercises will be explored in this meeting.

Data analysis to:

1. the current status of PFMT delivered in the hospital

2. perceived barriers and facilitators of delivering group-based PFMT to pregnant women

3. perceived barriers and facilitators of attending group-based PFMT programme

4. needs of pregnant women

Phase 2 and Phase 3

Publish findings of feasibility RCT

**Collect follow-up data (ICIQ-SF) at 42 days after delivery for both study groups**

**Collect data on 36 gestational weeks (ICIQ-SF for both study groups)**

**Collect baseline data**

From control group

**PHASE 2: Feasibility RCT**

**Flow for participants**

Recruit 48 participants to participate in feasibility study:

Randomise the participants to the group-based PFMT programme or the usual antenatal care

**PHASE 3:**

Interview with the 6-8 participants in the intervention group and the midwife who delivered the training session

**Refine the protocol for the group-based PFMT programme and definitive trial design**

Implement group-based PFMT supervision according to implementation plan

**Collect baseline data**

From intervention group

Qualitative & quantitative data analysis

**42-day after delivery:**

From control group: No one will be followed up

From intervention group:

No. lost follow-up + reasons if applicable

**37 gestational weeks:**

From intervention group:

The acceptability of the group-based PFMT programme

**Baseline:**

No. of participants:

-Invited to participate

-Excluded (not consenting/not met inclusion criteria/other reasons)

**37 gestational weeks:**

From control group:

No. lost to follow-up + reasons if applicable

From intervention group:

No. lost follow-up + reasons if applicable

No. discontinued intervention + reasons

**Data Collection – Feasibility Outcomes**
