# Supplementary material for: Group-based PFMT programme for preventing and/or treating UI in pregnant women: protocol of a randomized controlled feasibility study
Source: Pilot Feasibility Stud. 2023 Oct 31;9:180. doi: 10.1186/s40814-023-01410-2 (PMC10617193; doi:10.1186/s40814-023-01410-2)
Supplement: Supplementary file 3 — Additional file 3. PFMT leaflets and training diary. [file 40814_2023_1410_MOESM3_ESM.pdf]

南京市妇幼保健院
Nanjing Maternity and Child Health Care Hospital
南京医科大学附属妇产医院
Women's Hospital of Nanjing Medical University

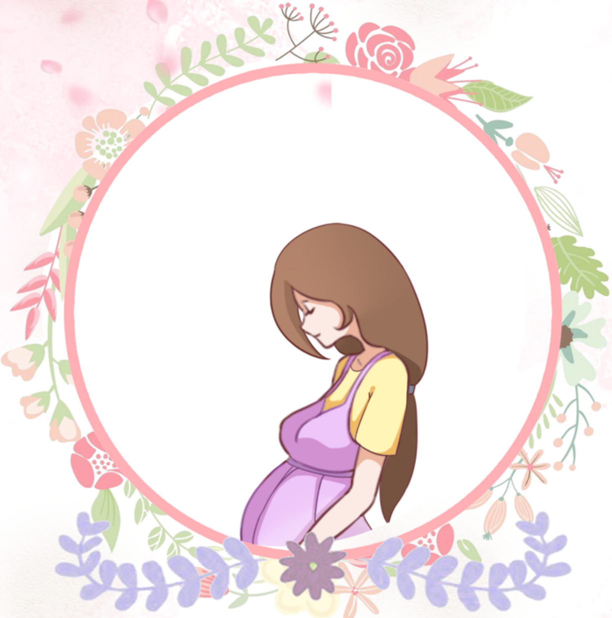

孕妇盆底肌锻炼指导手册

KING'S College LONDON

以群组的形式指导孕妇进行盆底肌锻炼

2022. 04

目 录

1. 什么是盆底肌？

2. 为什么要进行盆底肌锻炼？

3. 如何感知盆底肌？

4. 孕期如何进行盆底肌锻炼及锻炼注意事项

5. 锻炼日记

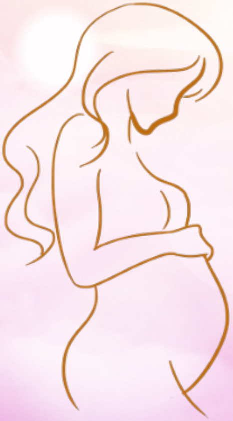

1. 什么是盆底肌？

盆底肌是指封闭骨盆底的肌肉群，这群肌肉如同一张“吊网”，将尿道、膀胱、阴道、子宫、直肠等器官紧紧兜住，从而使器官维持正常的位置发挥其功能。怀孕时子宫重量增加，盆底肌肉就持续受压而松弛，引发盆底疾病，如咳嗽、打喷嚏漏尿，阴道松弛，子宫脱垂，大便失禁等。

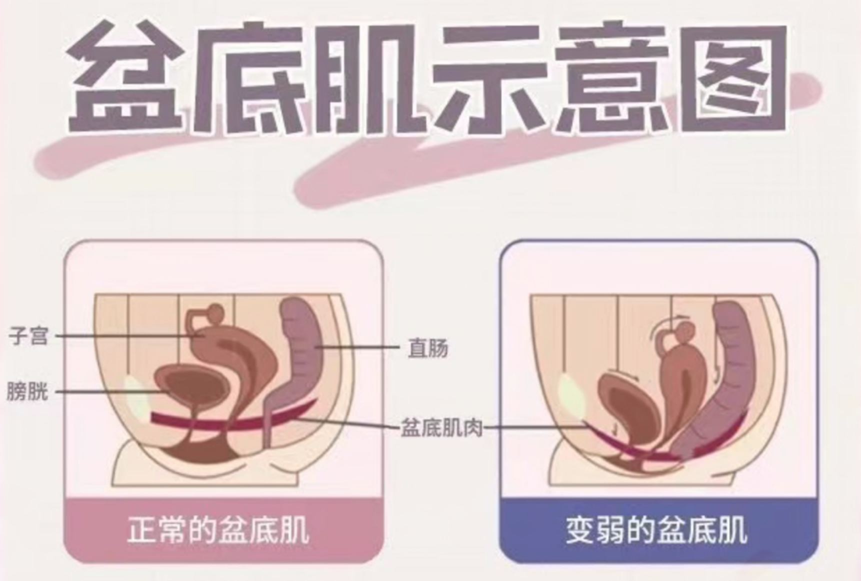

2. 为什么要进行盆底肌锻炼？

ACOG

THE AMERICAN CONGRESS OF OBSTETRICIANS AND GYNECOLOGISTS

美国妇产科学会（ACOG）认为在没有妊娠高血压、先兆早产、前置胎盘等妊娠期盆底肌肉训练禁忌症的情况下，应鼓励孕妇参加规律的盆底肌肌肉训练。

NICE National Institute for Health and Care Excellence

国际尿控协会（ICI）英国国家卫生和临床医疗优选研究所（NICE）建议孕妇进行盆底肌训练预防产后尿失禁。

中華醫學會 CHINESE MEDICAL ASSOCIATION

中华医学会妇产科学分会盆底学组建议孕妇进行盆底肌训练预防产后尿失禁。

3. 如何感知盆底肌？

在排尿的时候尝试暂停排尿，感受到收紧的部位就是盆底肌。（不建议反复做此动作，训练是在非排尿时段进行）

或者洗手后，将一根手指的两个指节放在阴道内，收紧阴道及肛门一带，感受到手指被周围肌肉裹紧，这部分肌肉就是盆底肌。

或者由专业人员通过观察阴道、肛门及会阴部的收缩判断盆底肌收缩是否正确。

2. 为什么要进行盆底肌锻炼？

有研究表明，妊娠 28 周（孕中后期）开始进行 Kegel 运动就可以明显缩短产程、提高顺产的机会。

另有研究表明，如果将孕期 Kegel 运动提前至妊娠 16 周（孕中期），结果发现坚持运动 8 周后与不进行包含 Kegel 在内的任何其他运动相比，也能有效缩短产程，大大减少产后漏尿的发生率。

医学综述发现孕期进行盆底肌锻炼可以改善盆底肌肌力、加速产程、促进阴道分娩；减少阴道分娩过程中会阴撕裂的发生；降低剖宫产率；预防和减少尿失禁、大便失禁的发生；预防和减少盆腔器官脱垂的发生；降低新生儿窒息率、降低产后出血率等。

参考文献：

1.邓姗，李晓玲．孕期盆底肌肉锻炼对孕妇产后盆底功能的影响[J]．中国当代医药, 2014, 21(21):57-59.

2.王建英，滕淑萍，曾小娥,等．产前盆底肌功能锻炼对产后康复的影响[J]．中华全科医学, 2010, 08(8):970-970.

3. National Institute for Health and Clinical Excellence (NICT). Urinary Incontinence: The Management of Urinary Incontinence in Women. NICE Clinical Guideline 40 (2006).

4. 卢明霞．妊娠期及产后早期盆底肌肉锻炼的研究进展[J]．医学综述, 2012, 18(24):4195-4197

4.孕期如何进行盆底肌锻炼及锻炼注意事项？

（1）准确地找到盆底肌的位置

（2）收缩盆底肌保持 6 秒钟后放松，重复 8 次后快速收缩、快速放松盆底肌 3-4 次，休息 2 分钟。共做 3 组训练，重复两次，但不要过度练习。

（3）练习时保持其他肌肉的放松，不要挤压或绷紧腹部、大腿、臀部的肌肉。

（4）姿势可随意选择，开始可以选择比较容易的姿势，如躺，逐渐可在坐姿或站姿下练习。

（5）要坚持每天锻炼，至少坚持 3 个月可以使盆底肌达到增强的效果。

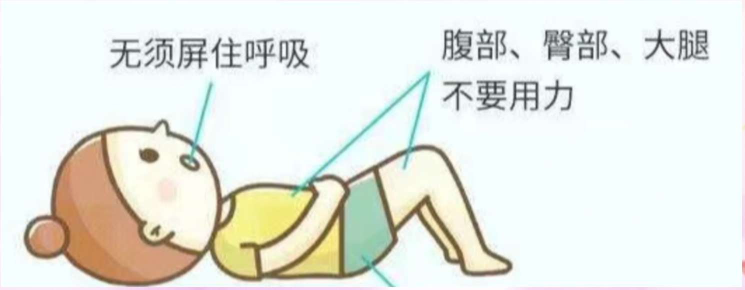

5. 锻炼日记

第一周

日期	第一次锻炼是否完成？	第一次锻炼完成组数	第二次锻炼是否完成？	第二次锻炼完成组数	今天是否漏尿？

第二周

日期	第一次锻炼是否完成？	第一次锻炼完成组数	第二次锻炼是否完成？	第二次锻炼完成组数	今天是否漏尿？

5. 锻炼日记

第三周

日期	第一次锻炼是否完成？	第一次锻炼完成组数	第二次锻炼是否完成？	第二次锻炼完成组数	今天是否漏尿？

第四周

日期	第一次锻炼是否完成？	第一次锻炼完成组数	第二次锻炼是否完成？	第二次锻炼完成组数	今天是否漏尿？

5. 锻炼日记

第五周

日期	第一次锻炼是否完成？	第一次锻炼完成组数	第二次锻炼是否完成？	第二次锻炼完成组数	今天是否漏尿？

第六周

日期	第一次锻炼是否完成？	第一次锻炼完成组数	第二次锻炼是否完成？	第二次锻炼完成组数	今天是否漏尿？

5. 锻炼日记

第七周

日期	第一次锻炼是否完成？	第一次锻炼完成组数	第二次锻炼是否完成？	第二次锻炼完成组数	今天是否漏尿？

第八周

日期	第一次锻炼是否完成？	第一次锻炼完成组数	第二次锻炼是否完成？	第二次锻炼完成组数	今天是否漏尿？

5. 锻炼日记

第九周

日期	第一次锻炼是否完成？	第一次锻炼完成组数	第二次锻炼是否完成？	第二次锻炼完成组数	今天是否漏尿？

第十周

日期	第一次锻炼是否完成？	第一次锻炼完成组数	第二次锻炼是否完成？	第二次锻炼完成组数	今天是否漏尿？

5. 锻炼日记

第十一周

日期	第一次锻炼是否完成？	第一次锻炼完成组数	第二次锻炼是否完成？	第二次锻炼完成组数	今天是否漏尿？

第十二周

日期	第一次锻炼是否完成？	第一次锻炼完成组数	第二次锻炼是否完成？	第二次锻炼完成组数	今天是否漏尿？

5. 锻炼日记

第十三周

日期	第一次锻炼是否完成？	第一次锻炼完成组数	第二次锻炼是否完成？	第二次锻炼完成组数	今天是否漏尿？

第十四周

日期	第一次锻炼是否完成？	第一次锻炼完成组数	第二次锻炼是否完成？	第二次锻炼完成组数	今天是否漏尿？

南京市妇幼保健院
Nanjing Maternity and Child Health Care Hospital
南京医科大学附属妇产医院
Women's Hospital of Nanjing Medical University

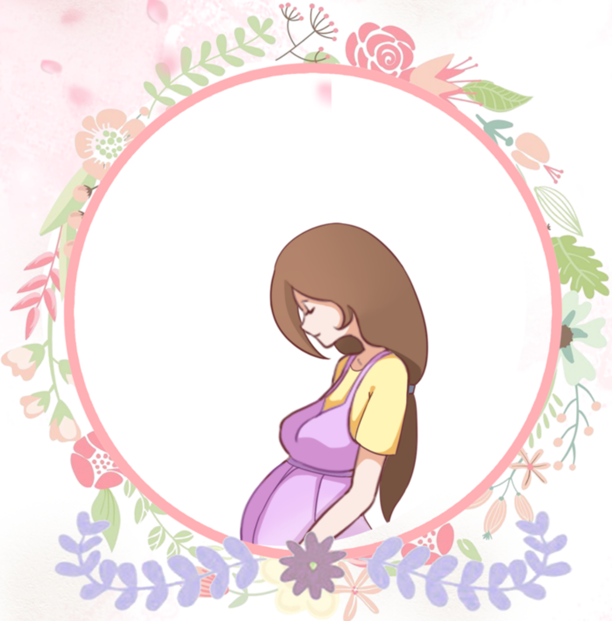

KING'S College LONDON

以群组的形式指导孕妇进行盆底肌锻炼

2022.04
