# Supplementary material for: Group-based PFMT programme for preventing and/or treating UI in pregnant women: protocol of a randomized controlled feasibility study
Source: Pilot Feasibility Stud. 2023 Oct 31;9:180. doi: 10.1186/s40814-023-01410-2 (PMC10617193; doi:10.1186/s40814-023-01410-2)
Supplement: Supplementary file 4 — Additional file 4: Table S1. Feasibility outcomes and progression criteria. [file 40814_2023_1410_MOESM4_ESM.docx]

| **Outcome** | **Objectives** | **Methods** | **Proceed with RCT** | **Amend- Proceed with changes** | **Stop- do not proceed unless changes are possible** |
| --- | --- | --- | --- | --- | --- |
| Recruitment rate | Assess the feasibility of recruiting eligible participants | Record the number of participants who received screening; record the reasons for participation refusal if possible | ≥50% of eligible participants consented to participate in the study in three months | ≥50% of eligible participants consented to participate in the study, but it takes longer than predicted | Unable to recruit participants |
| Retention rate | Assess the withdrawal rate during the sessions | Completion of baseline and follow-up questionnaires | ≥83% (40/48) of the participants submit the baseline questionnaires; ≥79% (38/48) of the participants submit the follow-up (37 gestational weeks) questionnaires and training diary;  ≥75% (36/48) of the participants submit the follow-up (42-day after delivery) questionnaires | ≥79% (38/48) of the participants submit the baseline questionnaires; ≥75% (36/48) of the participants submit the follow-up (37 gestational weeks) questionnaires and training diary;  ≥70% (34/48) of the participants submit the follow-up (42-day after delivery) questionnaires | <79% (38/48) of the participants submit the baseline questionnaires; <75% (36/48) of the participants submit the follow-up (37 gestational weeks) questionnaires and training diary;  <70% (34/48) of the participants submit the follow-up (42-day after delivery) questionnaires |
| Adherence | Evaluate the adherence rate of participants to the intervention | The registration of session attendance; The number of performing PFMT recorded in the training diary | ≥75% (18/24) of the participants complete at least 75% (3/4) of the face-to-face sessions; ≥75% (18/24) of the participants perform PFMT twice a day at home and record it on the training diary. | ≥62.5% (15/24) of the participants complete at least 75% (3/4) of the face-to-face sessions; ≥62.5% (15/24) of the participants perform PFMT twice a day at home and record it on the training diary. | <62.5% (15/24) of the participants complete at least 75% (3/4) of the face-to-face sessions; <62.5% (15/24) of the participants perform PFMT twice a day at home and record it on the training diary. |
| Acceptability of the intervention | Assess the participants’ experience of attending the intervention | Semi-structured interview with six participants in groups | Participants will have strongly positive views on their participation in the intervention. | Participants will have positive views on their participation in the intervention. | Negative views on their participation in the intervention. |
| Delivery | Assess the midwife’s view on the intervention delivery | Semi-structured interview with the midwife | The midwife will have strongly positive views on delivering the intervention. | The midwife will have positive views on delivering the intervention. | Negative views on their participation in the intervention. |

**Supplementary table 1 Feasibility outcomes and progression criteria**
